# Supplementary material for: Differential Infiltration of T-Cell Populations in Tumor and Liver Tissues Predicts Recurrence-Free Survival in Surgically Resected Hepatocellular Carcinoma
Source: Cancers (Basel). 2025 May 2;17(9):1548. doi: 10.3390/cancers17091548 (PMC12072143; doi:10.3390/cancers17091548)
Supplement: Supplementary file 1 [file cancers-17-01548-s001.zip › cancers-3625772-supplementary.pdf]

cancers

Article

Differential Infiltration of T-Cell Populations in Tumor and Liver Tissues Predicts Recurrence-Free Survival in Surgically Resected Hepatocellular Carcinoma

Eun Ji Jang [1,2], Ho Joong Choi [3], Young Kyoung You [3], Deok Hwa Seo [1,2], Mi Hyun Kwon [1,2], Keungmo Yang [2,4], Jaejun Lee [2,4], Jeong Won Jang [2,4], Seung Kew Yoon [2,4], Ji Won Han [1,2,4,*] and Pil Soo Sung [1,2,4,*]

Supplementary Materials

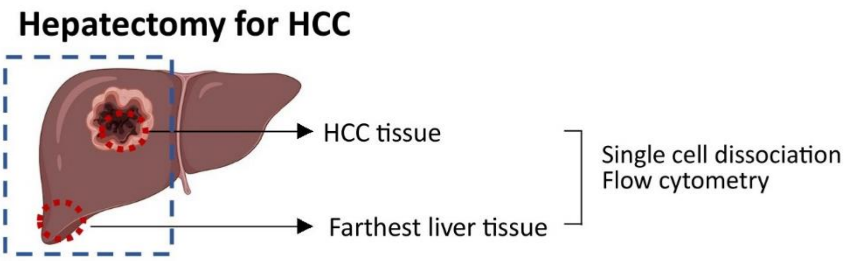

Figure S1. T-cell analysis in tumor and farthest non-tumor liver tissues from. patients with hepatocellular carcinoma (HCC).

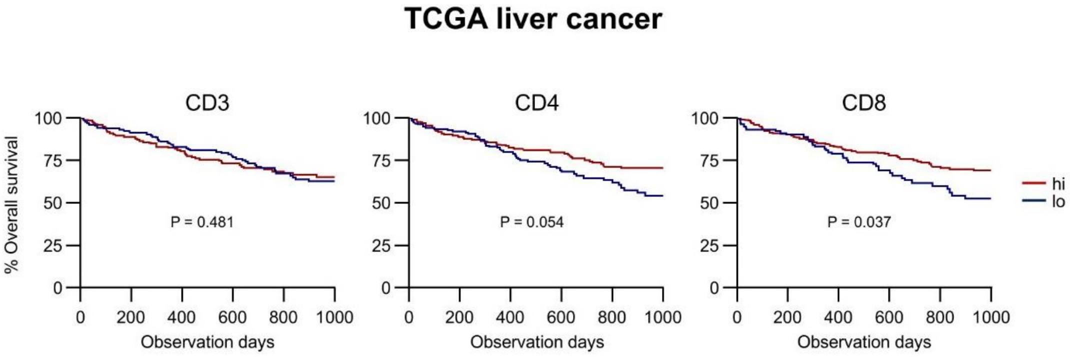

Figure S2. Overall survival analysis based on T-cell marker expression levels in patients with The Cancer Genome Atlas (TCGA) liver cancer.

 https://doi.org/10.3390/cancers17091548

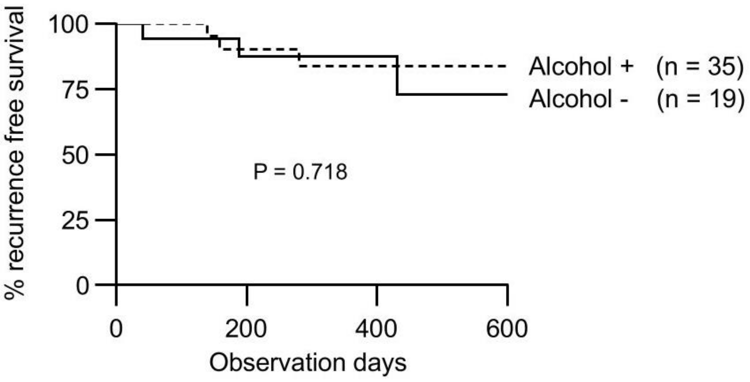

Figure S3. Recurrence-free survival (RFS) in patients with alcohol-related and non-alcohol-related liver disease.

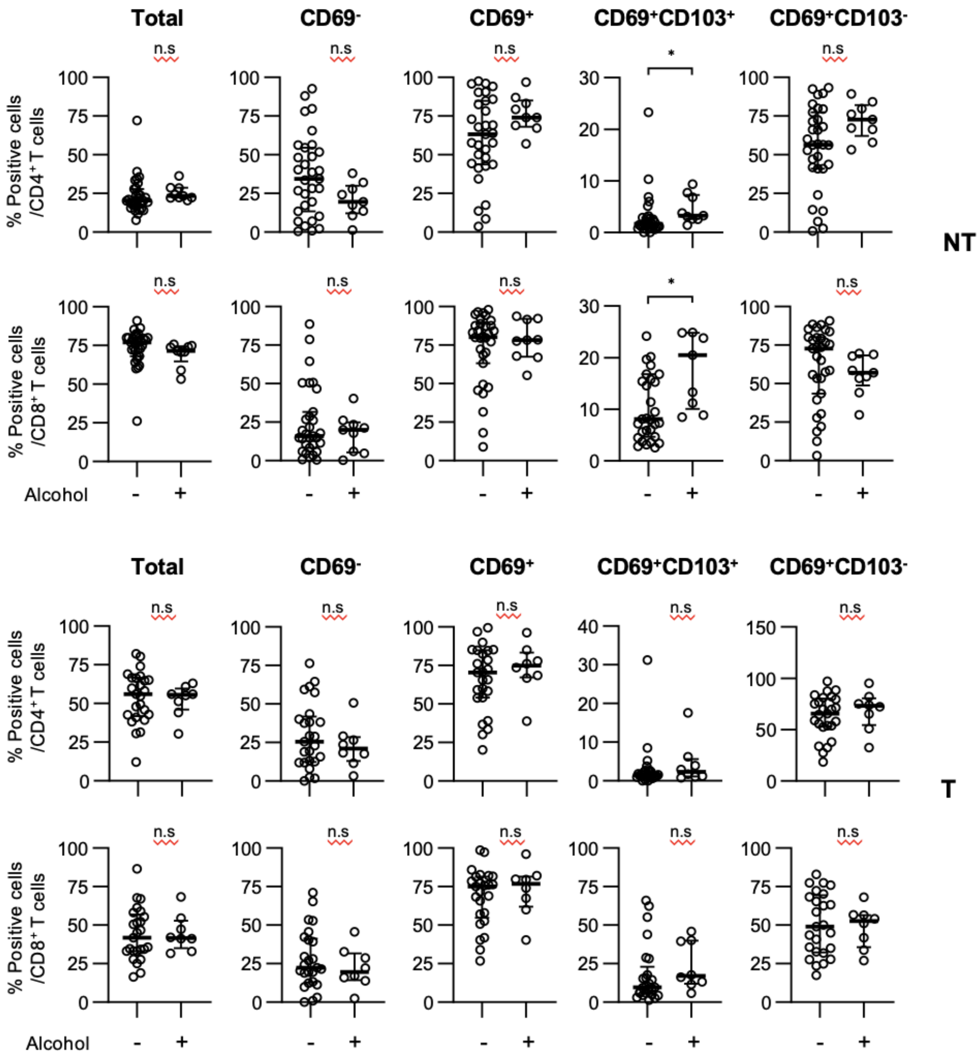

Figure S4. Specifically, the dataset was reanalyzed by categorizing 9 patients as ALD (without HBV co-infection) and 31 as non-ALD. While the overall patterns were maintained, the results for CD69⁻, CD69⁺, and CD69⁺CD103⁻ subsets of CD4⁺ T cells from both NT and T tissues, which were previously significant in Figure 2B and C, became non-significant after reanalysis. Statistical analysis was

performed using the Mann-Whitney U test. Statistical significance is indicated as follows: * $p < 0.05$, ** $p < 0.01$, **** $p < 0.0001$; n.s., not signifi-cant; NT, non-tumor tissue; T, tumor tissues; ALD, alcohol-related liver disease.

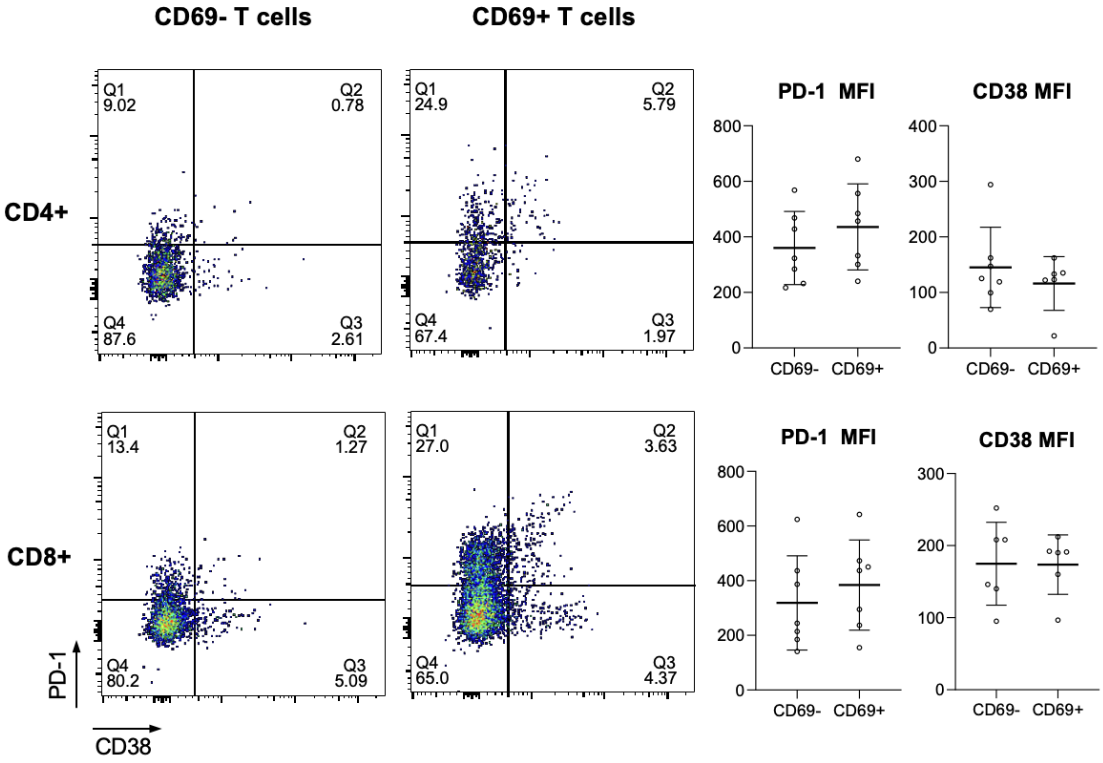

Figure S5. Staining and mean fluorescence intensity (MFI) analysis of CD69, PD-1, and CD38 in T cells. Statistical analysis was performed using the Mann-Whitney U test.
